# Supplementary material for: Glucose availability tunes latent CD8+ T cell expansion potential through a mitogen-independent, mTOR-dependent regulatory switch
Source: bioRxiv. 2026 Jan 17:2026.01.16.699963. Preprint. [Version 1] doi: 10.64898/2026.01.16.699963 (PMC12871354; doi:10.64898/2026.01.16.699963)
Supplement: 1 [file NIHPP2026.01.16.699963V1-supplement-1.pdf]

Supplement Figure 1

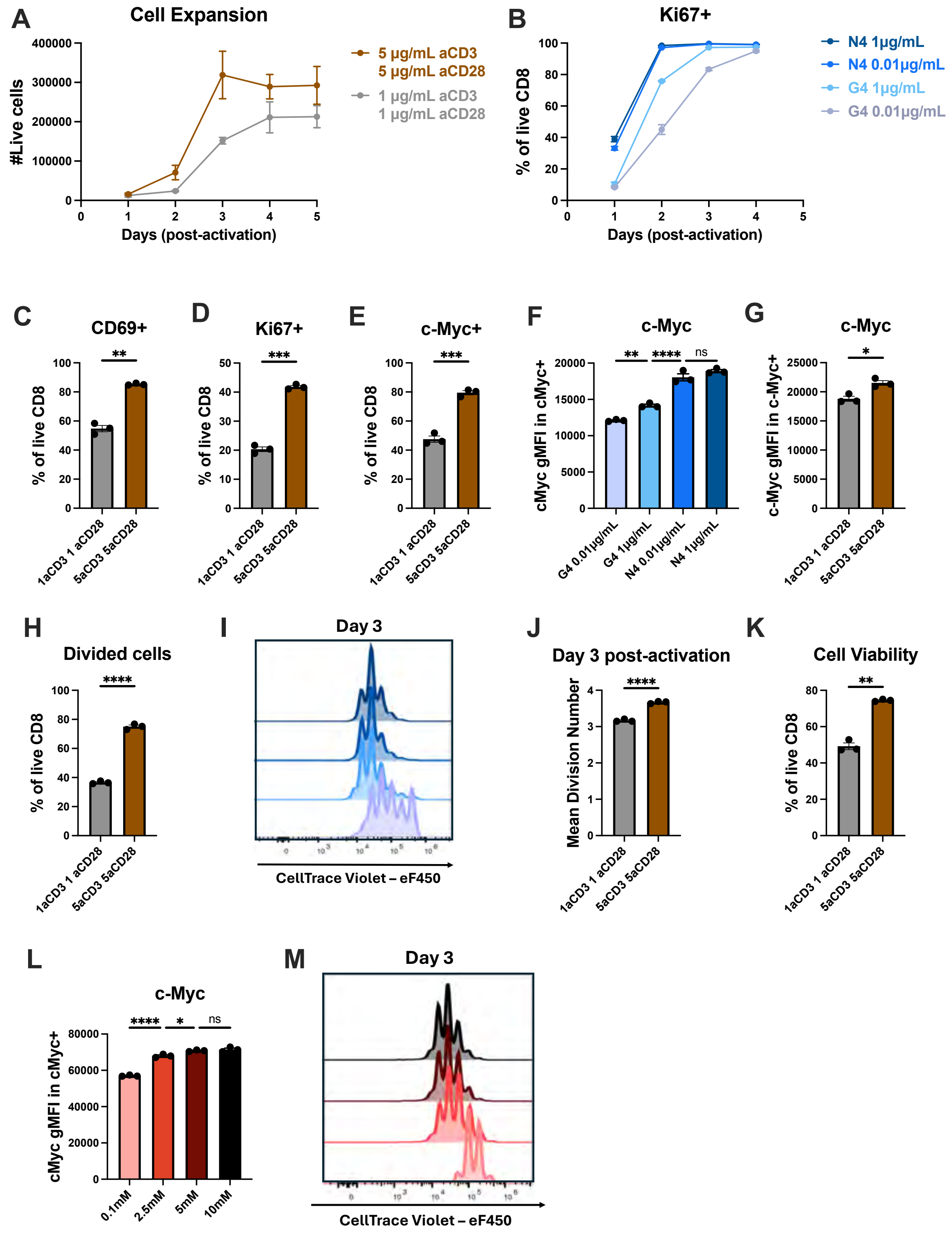

Supplement 1: CD8+ T cell proliferation is controlled by both the strength of TCR stimulation and environmental nutrients

CD8+ T cells were purified from OT-I spleen and lymph nodes that had been stimulated for 24 hours with either 5μg/mL or 1μg/mL of anti-CD3/CD28. (**A**) Quantification of number of live CD8+ T cells activated by different anti-CD3/CD28 concentrations. (**B**) Frequency of CD8+ T cells entering cell cycle (Ki67+) over 4 days after activation. (**C**) Frequency of CD8+ T cells expressing activation marker CD69 24 hours post-activation. (**D**) Frequency of CD8+ T cells entering cell cycle (Ki67+) 24 hours post-activation. (**E**) Frequency of CD8+ T cells expressing c-Myc 24 hours post-activation. (**F**) c-Myc geometric mean fluorescence intensity (gMFI) in CD8+ T cells expressing c-Myc 24 hours post-activation. (**G**) c-Myc gMFI in CD8+ T cells expressing c-Myc 24 hours post-activation. (**H**) Frequency of divided cells (≥ 1 division) on day 2 post-activation. (**I**) Representative histogram of CellTrace Violet (CTV) on day 3 post-activation. (**J**) Mean division number on day 3 post-activation. (**K**) Frequency of live CD8+ T cells 24 hours post-activation. (**L**) c-Myc gMFI in CD8+ T cells expressing c-Myc 24 hours post-activation. (**M)** Representative histogram of CTV on day 3 post-activation. All error bars are representative of 3 technical replicates. Statistical significance in (C, D, E, G, H, J, and K) was calculated using Welch's t-test, and statistical significance in (F and L) was calculated using one-way ANOVA with multiple comparisons and Tukey's correction.

Supplement Figure 2

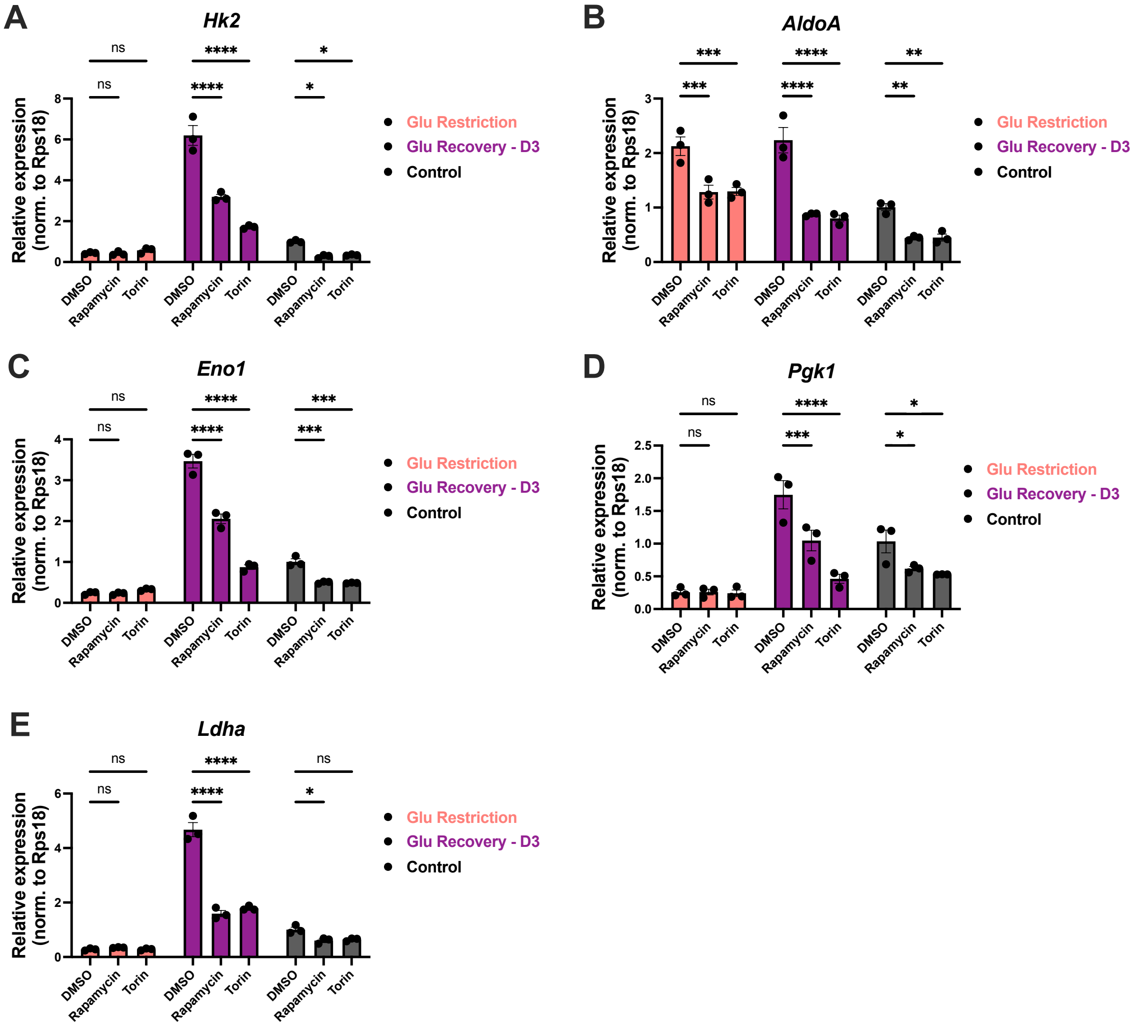

Supplement 2: mTORC signaling is essential for increased transcript abundance of key glycolytic enzymes

On day 3 post-activation, cells in their respective media conditions were treated with DMSO, rapamycin (50nM), or Torin 1 (50nM), and samples were collected on day 4 (24-hour treatment). Relative mRNA expression of key glycolytic enzymes (**A**) hexokinase 2 (*Hk2*), (**B**) aldolase (*AldolA*), (**C**) enolase 1 (*Eno1*), (**D**) phosphoglycerate kinase 1 (*Pgk1*), (**E**) lactate dehydrogenase A (*Ldha*). All error bars are representative of 3 technical replicates. Statistical significance was calculated using two-way ANOVA with multiple comparisons and Tukey's correction.
